# Supplementary material for: A Nanoporous Supramolecular Metal–Organic Framework Based on a Nucleotide: Interplay of the π···π Interactions Directing Assembly and Geometric Matching of Aromatic Tails
Source: Molecules. 2021 Jul 29;26(15):4594. doi: 10.3390/molecules26154594 (PMC8347718; doi:10.3390/molecules26154594)

checkCIF/PLATON report

Structure factors have been supplied for datablock(s) CuBr_CMP_SQ

THIS REPORT IS FOR GUIDANCE ONLY. IF USED AS PART OF A REVIEW PROCEDURE FOR PUBLICATION, IT SHOULD NOT REPLACE THE EXPERTISE OF AN EXPERIENCED CRYSTALLOGRAPHIC REFEREE.

No syntax errors found. CIF dictionary Interpreting this report

Datablock: CuBr_CMP_SQ

```
Bond precision:  C-C = 0.0250 A          Wavelength=0.71073

Cell:            a=31.9472(12)     b=31.9472(12)     c=11.3809(4)
                 alpha=90          beta=90           gamma=90
Temperature:     100 K
```

	Calculated	Reported
Volume	11615.6(10)	11615.6(10)
Space group	P 4	P 4
Hall group	P 4	P 4
Moiety formula	2(C78 H72 Br4 Cu6 N18 O19 P2), 4(Br), 31(O) [+ solvent]	?
Sum formula	C156 H144 Br12 Cu12 N36 O69 P4 [+ solvent]	C78 H164 Br6 Cu6 N18 O63 P2
Mr	5472.36	3284.90
Dx,g cm-3	1.565	1.878
Z	2	4
Mu (mm-1)	3.248	3.282
F000	5424.0	6704.0
F000'	5428.00	
h,k,lmax	40,40,14	39,39,14
Nref	23958[12609]	23831
Tmin,Tmax	0.681,0.720	0.811,0.982
Tmin'	0.668	

```
Correction method= # Reported T Limits: Tmin=0.811 Tmax=0.982
AbsCorr = MULTI-SCAN

Data completeness= 1.89/0.99      Theta(max)= 26.438

R(reflections)= 0.0891( 19068)    wR2(reflections)= 0.2417( 23831)

S = 1.048                   Npar= 1222
```

The following ALERTS were generated. Each ALERT has the format
test-name_ALERT_alert-type_alert-level.
Click on the hyperlinks for more details of the test.

Alert level A

```
PLAT213_ALERT_2_A Atom C6B            has ADP max/min Ratio .....        5.2 prolat
PLAT213_ALERT_2_A Atom C24            has ADP max/min Ratio .....        8.5 prolat
PLAT234_ALERT_4_A Large Hirshfeld Difference C23      --C24      .      0.35 Ang.
PLAT234_ALERT_4_A Large Hirshfeld Difference C55      --C56      .      0.35 Ang.
PLAT241_ALERT_2_A High   'MainMol' Ueq as Compared to Neighbors of      C2B' Check
PLAT375_ALERT_2_A Strange C-O-H Geometry (C-O  >   1.45 Ang) .....      O3B' Check
PLAT410_ALERT_2_A Short Intra H...H Contact  H1BA     ..H2BB     .      1.75 Ang.
                                                      x,y,z  =      1_555 Check
PLAT414_ALERT_2_A Short Intra D-H..H-X       H2BB     ..H2BA            1.74 Ang.
                                                      x,y,z  =      1_555 Check
PLAT430_ALERT_2_A Short Inter D...A Contact  O9W      ..O15W     .      2.29 Ang.
                                                      x,y,z  =      1_555 Check
```

Author Response: Highly disordered solvent molecules in huge pores

Alert level B

```
PLAT201_ALERT_2_B Isotropic non-H Atoms in Main Residue(s) .......         3 Report
              C1B'      C2B'      C28
```

Author Response: Residues with minor disordere, belonging to CMP ligand

```
PLAT213_ALERT_2_B Atom O3             has ADP max/min Ratio .....        4.9 prolat
PLAT213_ALERT_2_B Atom C53            has ADP max/min Ratio .....        4.1 prolat
PLAT213_ALERT_2_B Atom C54            has ADP max/min Ratio .....        4.2 prolat
PLAT220_ALERT_2_B NonSolvent   Resd 1  C   Ueq(max)/Ueq(min) Range      10.0 Ratio
```

Author Response: disorder

```
PLAT220_ALERT_2_B NonSolvent   Resd 1  O   Ueq(max)/Ueq(min) Range       7.4 Ratio
```

Author Response: disorder

```
PLAT234_ALERT_4_B Large Hirshfeld Difference O2B'     --C3B'     .      0.30 Ang.
PLAT234_ALERT_4_B Large Hirshfeld Difference N9       --C41      .      0.30 Ang.
PLAT234_ALERT_4_B Large Hirshfeld Difference C48      --C49      .      0.26 Ang.
PLAT241_ALERT_2_B High   'MainMol' Ueq as Compared to Neighbors of       C23 Check
PLAT241_ALERT_2_B High   'MainMol' Ueq as Compared to Neighbors of       C27 Check
PLAT241_ALERT_2_B High   'MainMol' Ueq as Compared to Neighbors of       C29 Check
PLAT241_ALERT_2_B High   'MainMol' Ueq as Compared to Neighbors of       C52 Check
PLAT242_ALERT_2_B Low    'MainMol' Ueq as Compared to Neighbors of      C3B' Check
```

Author Response: Thermal disorder

PLAT242_ALERT_2_B Low   'MainMol' Ueq as Compared to Neighbors of   C22 Check

Author Response: Thermal disorder

PLAT242_ALERT_2_B Low   'MainMol' Ueq as Compared to Neighbors of   C28 Check

Author Response: Thermal disorder

PLAT306_ALERT_2_B Isolated Oxygen Atom (H-atoms Missing ?) ....   O3W Check

Author Response: Hydrogen atoms on solvent molecules were neither found nor calculated

PLAT306_ALERT_2_B Isolated Oxygen Atom (H-atoms Missing ?) ....   O4W Check

Author Response: Hydrogen atoms on solvent molecules were neither found nor calculated

PLAT306_ALERT_2_B Isolated Oxygen Atom (H-atoms Missing ?) ....   O5W Check

Author Response: Hydrogen atoms on solvent molecules were neither found nor calculated

PLAT306_ALERT_2_B Isolated Oxygen Atom (H-atoms Missing ?) ....   O6W Check

Author Response: Hydrogen atoms on solvent molecules were neither found nor calculated

PLAT306_ALERT_2_B Isolated Oxygen Atom (H-atoms Missing ?) ....   O7W Check

Author Response: Hydrogen atoms on solvent molecules were neither found nor calculated

PLAT306_ALERT_2_B Isolated Oxygen Atom (H-atoms Missing ?) ....   O8W Check

Author Response: Hydrogen atoms on solvent molecules were neither found nor calculated

PLAT306_ALERT_2_B Isolated Oxygen Atom (H-atoms Missing ?) ....   O9W Check

Author Response: Hydrogen atoms on solvent molecules were neither found nor calculated

PLAT306_ALERT_2_B Isolated Oxygen Atom (H-atoms Missing ?) ....   O11W Check

Author Response: Hydrogen atoms on solvent molecules were neither found nor calculated

```
PLAT306_ALERT_2_B Isolated Oxygen Atom (H-atoms Missing ?) .......       O12W Check
```

Author Response: Hydrogen atoms on solvent molecules were neither found nor calculated

```
PLAT306_ALERT_2_B Isolated Oxygen Atom (H-atoms Missing ?) .......       O14W Check
```

Author Response: Hydrogen atoms on solvent molecules were neither found nor calculated

```
PLAT306_ALERT_2_B Isolated Oxygen Atom (H-atoms Missing ?) .......       O16W Check
```

Author Response: Hydrogen atoms on solvent molecules were neither found nor calculated

```
PLAT306_ALERT_2_B Isolated Oxygen Atom (H-atoms Missing ?) .......       O18W Check
```

Author Response: Hydrogen atoms on solvent molecules were neither found nor calculated

```
PLAT306_ALERT_2_B Isolated Oxygen Atom (H-atoms Missing ?) .......       O19W Check
```

Author Response: Hydrogen atoms on solvent molecules were neither found nor calculated

```
PLAT306_ALERT_2_B Isolated Oxygen Atom (H-atoms Missing ?) .......       O20W Check
```

Author Response: Hydrogen atoms on solvent molecules were neither found nor calculated

```
PLAT306_ALERT_2_B Isolated Oxygen Atom (H-atoms Missing ?) .......       O21W Check
```

Author Response: Hydrogen atoms on solvent molecules were neither found nor calculated

```
PLAT306_ALERT_2_B Isolated Oxygen Atom (H-atoms Missing ?) .......       O22W Check
```

Author Response: Hydrogen atoms on solvent molecules were neither found nor calculated

```
PLAT341_ALERT_3_B Low Bond Precision on  C-C Bonds ...............    0.02505 Ang.
```

Author Response: likely due to disorder

```
PLAT420_ALERT_2_B D-H Bond Without Acceptor  O2A'     --H2AB     .     Please Check
```

Author Response: Likely due to absence of hydrogen atoms on solvent molecules that were neither found nor calculated

```
PLAT430_ALERT_2_B Short Inter D...A Contact  O2B      ..O22W     .       2.84 Ang.
                                                    1-y,x,z  =      3_655 Check
```

Author Response: Highly disordered solvent molecules in huge pores

```
PLAT430_ALERT_2_B Short Inter D...A Contact  O11W     ..O12W     .       2.70 Ang.
                                                      x,y,z  =      1_555 Check
```

Author Response: Highly disordered solvent molecules in huge pores

```
PLAT430_ALERT_2_B Short Inter D...A Contact  O14W     ..O22W     .       2.80 Ang.
                                                 1-y,x,-1+z  =      3_654 Check
```

Author Response: Highly disordered solvent molecules in huge pores

```
PLAT430_ALERT_2_B Short Inter D...A Contact  O14W     ..O14W     .       2.81 Ang.
                                                    y,1-x,z  =      4_565 Check
```

Author Response: Highly disordered solvent molecules in huge pores

```
PLAT430_ALERT_2_B Short Inter D...A Contact  O14W     ..O14W     .       2.81 Ang.
                                                    1-y,x,z  =      3_655 Check
```

Author Response: Highly disordered solvent molecules in huge pores

```
PLAT430_ALERT_2_B Short Inter D...A Contact  O18W     ..O22W     .       2.71 Ang.
                                                      x,y,z  =      1_555 Check
```

Author Response: Highly disordered solvent molecules in huge pores

```
PLAT430_ALERT_2_B Short Inter D...A Contact  O18W     ..O19W     .       2.72 Ang.
                                                      x,y,z  =      1_555 Check
```

Author Response: Highly disordered solvent molecules in huge pores

```
PLAT430_ALERT_2_B Short Inter D...A Contact  O20W     ..O22W     .       2.79 Ang.
                                                  1-x,1-y,z  =      2_665 Check
```

Author Response: Highly disordered solvent molecules in huge pores

```
PLAT987_ALERT_1_B The Flack x is >> 0 -  Do a BASF/TWIN Refinement     Please Check
```

Author Response: The Flack value is 0.089(4), very close to zero

```
PLAT990_ALERT_1_B Deprecated .res/.hkl Input Style SQUEEZE Job ...          ! Note
```

Author Response: No ABIN command used, but all details have been attached to CIF

Alert level C

```
PLAT202_ALERT_3_C Isotropic non-H Atoms in Anion/Solvent .........          2 Check
              O21W    O22W
PLAT213_ALERT_2_C Atom O2B'           has ADP max/min Ratio .....        3.8 prolat
PLAT213_ALERT_2_C Atom N5             has ADP max/min Ratio .....        3.8 prolat
PLAT213_ALERT_2_C Atom N6             has ADP max/min Ratio .....        3.3 prolat
PLAT213_ALERT_2_C Atom C3B'           has ADP max/min Ratio .....        3.7 prolat
PLAT213_ALERT_2_C Atom C17            has ADP max/min Ratio .....        3.3 prolat
PLAT213_ALERT_2_C Atom C23            has ADP max/min Ratio .....        3.9 prolat
PLAT213_ALERT_2_C Atom C26            has ADP max/min Ratio .....        3.2 prolat
PLAT213_ALERT_2_C Atom C27            has ADP max/min Ratio .....        3.9 prolat
PLAT213_ALERT_2_C Atom C29            has ADP max/min Ratio .....        3.1 prolat
PLAT213_ALERT_2_C Atom C30            has ADP max/min Ratio .....        3.3 prolat
PLAT213_ALERT_2_C Atom C41            has ADP max/min Ratio .....        3.8 prolat
PLAT213_ALERT_2_C Atom C45            has ADP max/min Ratio .....        3.5 prolat
PLAT213_ALERT_2_C Atom C47            has ADP max/min Ratio .....        3.4 prolat
PLAT213_ALERT_2_C Atom C52            has ADP max/min Ratio .....        3.7 prolat
PLAT213_ALERT_2_C Atom C55            has ADP max/min Ratio .....        3.9 prolat
PLAT213_ALERT_2_C Atom C57            has ADP max/min Ratio .....        3.8 prolat
PLAT213_ALERT_2_C Atom C58            has ADP max/min Ratio .....        3.6 prolat
PLAT213_ALERT_2_C Atom C59            has ADP max/min Ratio .....        3.7 prolat
PLAT222_ALERT_3_C NonSolvent Resd 1  H   Uiso(max)/Uiso(min) Range       10.0 Ratio
PLAT234_ALERT_4_C Large Hirshfeld Difference O4B'     --C4B'     .       0.18 Ang.
PLAT234_ALERT_4_C Large Hirshfeld Difference N1B      --C6B      .       0.21 Ang.
PLAT234_ALERT_4_C Large Hirshfeld Difference N7       --C31      .       0.16 Ang.
PLAT234_ALERT_4_C Large Hirshfeld Difference N10      --C46      .       0.20 Ang.
PLAT234_ALERT_4_C Large Hirshfeld Difference N12      --C60      .       0.24 Ang.
PLAT234_ALERT_4_C Large Hirshfeld Difference C5A      --C6A      .       0.17 Ang.
PLAT234_ALERT_4_C Large Hirshfeld Difference C7       --C8       .       0.17 Ang.
PLAT234_ALERT_4_C Large Hirshfeld Difference C14      --C15      .       0.20 Ang.
PLAT234_ALERT_4_C Large Hirshfeld Difference C15      --C16      .       0.21 Ang.
PLAT234_ALERT_4_C Large Hirshfeld Difference C17      --C18      .       0.23 Ang.
PLAT234_ALERT_4_C Large Hirshfeld Difference C25      --C26      .       0.22 Ang.
PLAT234_ALERT_4_C Large Hirshfeld Difference C32      --C33      .       0.19 Ang.
PLAT234_ALERT_4_C Large Hirshfeld Difference C33      --C34      .       0.24 Ang.
PLAT234_ALERT_4_C Large Hirshfeld Difference C37      --C38      .       0.20 Ang.
PLAT234_ALERT_4_C Large Hirshfeld Difference C42      --C43      .       0.22 Ang.
PLAT234_ALERT_4_C Large Hirshfeld Difference C47      --C48      .       0.25 Ang.
PLAT241_ALERT_2_C High   'MainMol' Ueq as Compared to Neighbors of        O1H Check
PLAT241_ALERT_2_C High   'MainMol' Ueq as Compared to Neighbors of        C41 Check
PLAT241_ALERT_2_C High   'MainMol' Ueq as Compared to Neighbors of        C54 Check
PLAT241_ALERT_2_C High   'MainMol' Ueq as Compared to Neighbors of        C58 Check
PLAT241_ALERT_2_C High   'MainMol' Ueq as Compared to Neighbors of        C59 Check
PLAT242_ALERT_2_C Low    'MainMol' Ueq as Compared to Neighbors of       O4B' Check
```

Author Response: Thermal disorder

```
PLAT242_ALERT_2_C Low    'MainMol' Ueq as Compared to Neighbors of        N1B Check
```

Author Response: Thermal disorder

```
PLAT242_ALERT_2_C Low    ’MainMol’ Ueq as Compared to Neighbors of       C1B’ Check
```

Author Response: Thermal disorder

```
PLAT242_ALERT_2_C Low    ’MainMol’ Ueq as Compared to Neighbors of        C2B Check
```

Author Response: Thermal disorder

```
PLAT242_ALERT_2_C Low    ’MainMol’ Ueq as Compared to Neighbors of       C4B’ Check
```

Author Response: Thermal disorder

```
PLAT242_ALERT_2_C Low    ’MainMol’ Ueq as Compared to Neighbors of        C25 Check
```

Author Response: Thermal disorder

```
PLAT242_ALERT_2_C Low    ’MainMol’ Ueq as Compared to Neighbors of        C51 Check
```

Author Response: Thermal disorder

```
PLAT242_ALERT_2_C Low    ’MainMol’ Ueq as Compared to Neighbors of        C53 Check
```

Author Response: Thermal disorder

```
PLAT242_ALERT_2_C Low    ’MainMol’ Ueq as Compared to Neighbors of        C60 Check
```

Author Response: Thermal disorder

```
PLAT260_ALERT_2_C Large Average Ueq of Residue Including       O7W      0.177 Check
PLAT260_ALERT_2_C Large Average Ueq of Residue Including       O8W      0.125 Check
PLAT260_ALERT_2_C Large Average Ueq of Residue Including       O9W      0.133 Check
PLAT260_ALERT_2_C Large Average Ueq of Residue Including      O11W      0.144 Check
PLAT260_ALERT_2_C Large Average Ueq of Residue Including      O12W      0.113 Check
PLAT260_ALERT_2_C Large Average Ueq of Residue Including      O14W      0.120 Check
PLAT260_ALERT_2_C Large Average Ueq of Residue Including      O16W      0.113 Check
PLAT260_ALERT_2_C Large Average Ueq of Residue Including      O18W      0.208 Check
PLAT260_ALERT_2_C Large Average Ueq of Residue Including      O19W      0.113 Check
PLAT260_ALERT_2_C Large Average Ueq of Residue Including      O20W      0.159 Check
PLAT360_ALERT_2_C Short  C(sp3)-C(sp3) Bond  C1B’     - C2B’      .        1.42 Ang.
PLAT360_ALERT_2_C Short  C(sp3)-C(sp3) Bond  C2B’     - C3B’      .        1.42 Ang.
PLAT360_ALERT_2_C Short  C(sp3)-C(sp3) Bond  C3B’     - C4B’      .        1.42 Ang.
PLAT368_ALERT_2_C Short  C(sp2)-C(sp2) Bond  C5B      - C6B       .        1.22 Ang.
PLAT414_ALERT_2_C Short Intra D-H..H-X       H2BB     ..H3BB               1.97 Ang.
                                                      x,y,z  =      1_555 Check
PLAT430_ALERT_2_C Short Inter D...A Contact  O4A’     ..O12W      .        2.88 Ang.
                                                      x,y,z  =      1_555 Check
```

Author Response: Highly disordered solvent molecules in huge pores

```
PLAT430_ALERT_2_C Short Inter D...A Contact  O11W     ..O16W     .       2.86 Ang.
                                                    x,y,z  =      1_555 Check
```

Author Response: Highly disordered solvent molecules in huge pores

```
PLAT430_ALERT_2_C Short Inter D...A Contact  O15W     ..O18W     .       2.85 Ang.
                                                  1-y,x,z  =      3_655 Check
```

Author Response: Highly disordered solvent molecules in huge pores

```
PLAT431_ALERT_2_C Short Inter HL..A Contact  Br5      ..O1W      .       3.25 Ang.
                                                    x,y,z  =      1_555 Check
PLAT906_ALERT_3_C Large K Value in the Analysis of Variance ......      3.741 Check
PLAT911_ALERT_3_C Missing FCF Refl Between Thmin & STh/L=    0.600          5 Report
PLAT918_ALERT_3_C Reflection(s) with I(obs) much Smaller I(calc) .          4 Check
PLAT934_ALERT_3_C Number of (Iobs-Icalc)/Sigma(W) > 10 Outliers ..          1 Check
PLAT974_ALERT_2_C Check Calcd Negative Resid. Density on       Cu1      -1.01 eA-3
PLAT975_ALERT_2_C Check Calcd Resid. Dens.  1.04A   From O3W          1.29 eA-3
PLAT975_ALERT_2_C Check Calcd Resid. Dens.  0.98A   From O6W          1.27 eA-3
PLAT975_ALERT_2_C Check Calcd Resid. Dens.  0.96A   From O6W          1.08 eA-3
PLAT975_ALERT_2_C Check Calcd Resid. Dens.  0.45A   From O21W         1.06 eA-3
PLAT975_ALERT_2_C Check Calcd Resid. Dens.  1.06A   From O5W          1.05 eA-3
PLAT975_ALERT_2_C Check Calcd Resid. Dens.  0.97A   From O3W          0.95 eA-3
PLAT975_ALERT_2_C Check Calcd Resid. Dens.  0.70A   From O13W         0.89 eA-3
PLAT975_ALERT_2_C Check Calcd Resid. Dens.  0.89A   From O13W         0.85 eA-3
PLAT976_ALERT_2_C Check Calcd Resid. Dens.  1.01A   From N8          -0.78 eA-3
PLAT977_ALERT_2_C Check Negative Difference Density on H5BA          -0.65 eA-3
PLAT977_ALERT_2_C Check Negative Difference Density on H5BB          -0.38 eA-3
PLAT977_ALERT_2_C Check Negative Difference Density on H6BA          -0.34 eA-3
PLAT977_ALERT_2_C Check Negative Difference Density on H2AA          -0.32 eA-3
PLAT977_ALERT_2_C Check Negative Difference Density on H34A          -0.33 eA-3
PLAT977_ALERT_2_C Check Negative Difference Density on H40A          -0.31 eA-3
```

Alert level G

```
FORMU01_ALERT_2_G  There is a discrepancy between the atom counts in the
            _chemical_formula_sum and the formula from the _atom_site* data.
            Atom count from _chemical_formula_sum:C78 H164 Br6 Cu6 N18 O63 P2
            Atom count from the _atom_site data:  C78 H72 Br6 Cu6 N18 O34.5 P2
CELLZ01_ALERT_1_G Difference between formula and atom_site contents detected.
CELLZ01_ALERT_1_G ALERT: Large difference may be due to a
            symmetry error - see SYMMG tests
           From the CIF: _cell_formula_units_Z    4
           From the CIF: _chemical_formula_sum  C78 H164 Br6 Cu6 N18 O63 P2
           TEST: Compare cell contents of formula and atom_site data

           atom    Z*formula  cif sites diff
           C        312.00    312.00    0.00
           H        656.00    288.00  368.00
           Br        24.00     24.00    0.00
           Cu        24.00     24.00    0.00
           N         72.00     72.00    0.00
           O        252.00    138.00  114.00
           P          8.00      8.00    0.00
PLAT007_ALERT_5_G Number of Unrefined Donor-H Atoms .............          8 Report
PLAT033_ALERT_4_G Flack x Value Deviates > 3.0 * sigma from Zero .      0.089 Note
PLAT041_ALERT_1_G Calc. and Reported SumFormula    Strings  Differ     Please Check
PLAT045_ALERT_1_G Calculated and Reported Z Differ by a Factor ...       0.50 Check
PLAT051_ALERT_1_G Mu(calc) and Mu(CIF) Ratio Differs from 1.0 by .       1.04 %
```

```
PLAT083_ALERT_2_G SHELXL Second Parameter in WGHT  Unusually Large      61.08 Why ?
PLAT232_ALERT_2_G Hirshfeld Test Diff (M-X)  Br4      --Cu6      .        8.6 s.u.
PLAT300_ALERT_4_G Atom Site Occupancy of O13W       Constrained at        0.5 Check
PLAT300_ALERT_4_G Atom Site Occupancy of O15W       Constrained at        0.5 Check
PLAT302_ALERT_4_G Anion/Solvent/Minor-Residue Disorder (Resd 13  )       100% Note
PLAT302_ALERT_4_G Anion/Solvent/Minor-Residue Disorder (Resd 15  )       100% Note
PLAT304_ALERT_4_G Non-Integer Number of Atoms in ..... (Resd 13  )       0.50 Check
PLAT304_ALERT_4_G Non-Integer Number of Atoms in ..... (Resd 15  )       0.50 Check
PLAT304_ALERT_4_G Non-Integer Number of Atoms in ..... (Resd 18  )       0.25 Check
PLAT304_ALERT_4_G Non-Integer Number of Atoms in ..... (Resd 20  )       0.25 Check
PLAT311_ALERT_2_G Isolated Disordered Oxygen Atom (No H’s ?) .....       O13W Check
PLAT311_ALERT_2_G Isolated Disordered Oxygen Atom (No H’s ?) .....       O15W Check
PLAT343_ALERT_2_G Unusual sp3      Angle Range in Main Residue for       C2B’ Check
PLAT398_ALERT_2_G Deviating  C-O-C    Angle From 120 for O4B’          108.1 Degree
PLAT432_ALERT_2_G Short Inter X...Y Contact  O15W     ..C2B’            2.29 Ang.
                                                      x,y,z  =      1_555 Check
PLAT432_ALERT_2_G Short Inter X...Y Contact  O15W     ..C1B’            2.53 Ang.
                                                      x,y,z  =      1_555 Check
PLAT432_ALERT_2_G Short Inter X...Y Contact  O15W     ..C3B’            2.55 Ang.
                                                      x,y,z  =      1_555 Check
PLAT432_ALERT_2_G Short Inter X...Y Contact  O15W     ..C4B’            2.91 Ang.
                                                      x,y,z  =      1_555 Check
PLAT606_ALERT_4_G Solvent Accessible VOID(S) in Structure ........          ! Info
PLAT720_ALERT_4_G Number of Unusual/Non-Standard Labels ..........         40 Note
PLAT790_ALERT_4_G Centre of Gravity not Within Unit Cell: Resd.  #          4 Note
              O
PLAT791_ALERT_4_G Model has Chirality at P1          (Sohnke SpGr)          R Verify
PLAT791_ALERT_4_G Model has Chirality at P2          (Sohnke SpGr)          R Verify
PLAT791_ALERT_4_G Model has Chirality at C1B’        (Sohnke SpGr)          R Verify
PLAT791_ALERT_4_G Model has Chirality at C2B’        (Sohnke SpGr)          S Verify
PLAT791_ALERT_4_G Model has Chirality at C3B’        (Sohnke SpGr)          S Verify
PLAT791_ALERT_4_G Model has Chirality at C4B’        (Sohnke SpGr)          R Verify
PLAT791_ALERT_4_G Model has Chirality at C3A’        (Sohnke SpGr)          S Verify
PLAT791_ALERT_4_G Model has Chirality at C4A’        (Sohnke SpGr)          R Verify
PLAT791_ALERT_4_G Model has Chirality at C2A’        (Sohnke SpGr)          R Verify
PLAT791_ALERT_4_G Model has Chirality at C1A’        (Sohnke SpGr)          R Verify
PLAT794_ALERT_5_G Tentative Bond Valency for Cu1       (II)      .       2.13 Info
PLAT794_ALERT_5_G Tentative Bond Valency for Cu2       (II)      .       2.14 Info
PLAT794_ALERT_5_G Tentative Bond Valency for Cu3       (II)      .       2.09 Info
PLAT794_ALERT_5_G Tentative Bond Valency for Cu4       (II)      .       2.18 Info
PLAT794_ALERT_5_G Tentative Bond Valency for Cu5       (II)      .       1.93 Info
PLAT794_ALERT_5_G Tentative Bond Valency for Cu6       (II)      .       1.96 Info
PLAT869_ALERT_4_G ALERTS Related to the Use of SQUEEZE Suppressed           ! Info
PLAT883_ALERT_1_G No Info/Value for _atom_sites_solution_primary .     Please Do !
PLAT910_ALERT_3_G Missing # of FCF Reflection(s) Below Theta(Min).          1 Note
PLAT912_ALERT_4_G Missing # of FCF Reflections Above STh/L=  0.600         49 Note
PLAT961_ALERT_5_G Dataset Contains no Negative Intensities .......     Please Check
PLAT965_ALERT_2_G The SHELXL WEIGHT Optimisation has not Converged     Please Check
PLAT978_ALERT_2_G Number C-C Bonds with Positive Residual Density.          0 Info
```

---

9 **ALERT level A** = Most likely a serious problem - resolve or explain
44 **ALERT level B** = A potentially serious problem, consider carefully
89 **ALERT level C** = Check. Ensure it is not caused by an omission or oversight
52 **ALERT level G** = General information/check it is not something unexpected

8 ALERT type 1 CIF construction/syntax error, inconsistent or missing data
125 ALERT type 2 Indicator that the structure model may be wrong or deficient
8 ALERT type 3 Indicator that the structure quality may be low
45 ALERT type 4 Improvement, methodology, query or suggestion
8 ALERT type 5 Informative message, check

It is advisable to attempt to resolve as many as possible of the alerts in all categories. Often the minor alerts point to easily fixed oversights, errors and omissions in your CIF or refinement strategy, so attention to these fine details can be worthwhile. In order to resolve some of the more serious problems it may be necessary to carry out additional measurements or structure refinements. However, the purpose of your study may justify the reported deviations and the more serious of these should normally be commented upon in the discussion or experimental section of a paper or in the "special_details" fields of the CIF. checkCIF was carefully designed to identify outliers and unusual parameters, but every test has its limitations and alerts that are not important in a particular case may appear. Conversely, the absence of alerts does not guarantee there are no aspects of the results needing attention. It is up to the individual to critically assess their own results and, if necessary, seek expert advice.

Publication of your CIF in IUCr journals

A basic structural check has been run on your CIF. These basic checks will be run on all CIFs submitted for publication in IUCr journals (*Acta Crystallographica*, *Journal of Applied Crystallography*, *Journal of Synchrotron Radiation*); however, if you intend to submit to *Acta Crystallographica Section C* or *E* or *IUCrData*, you should make sure that full publication checks are run on the final version of your CIF prior to submission.

Publication of your CIF in other journals

Please refer to the *Notes for Authors* of the relevant journal for any special instructions relating to CIF submission.

PLATON version of 03/06/2021; check.def file version of 02/06/2021

Datablock CuBr_CMP_SQ - ellipsoid plot

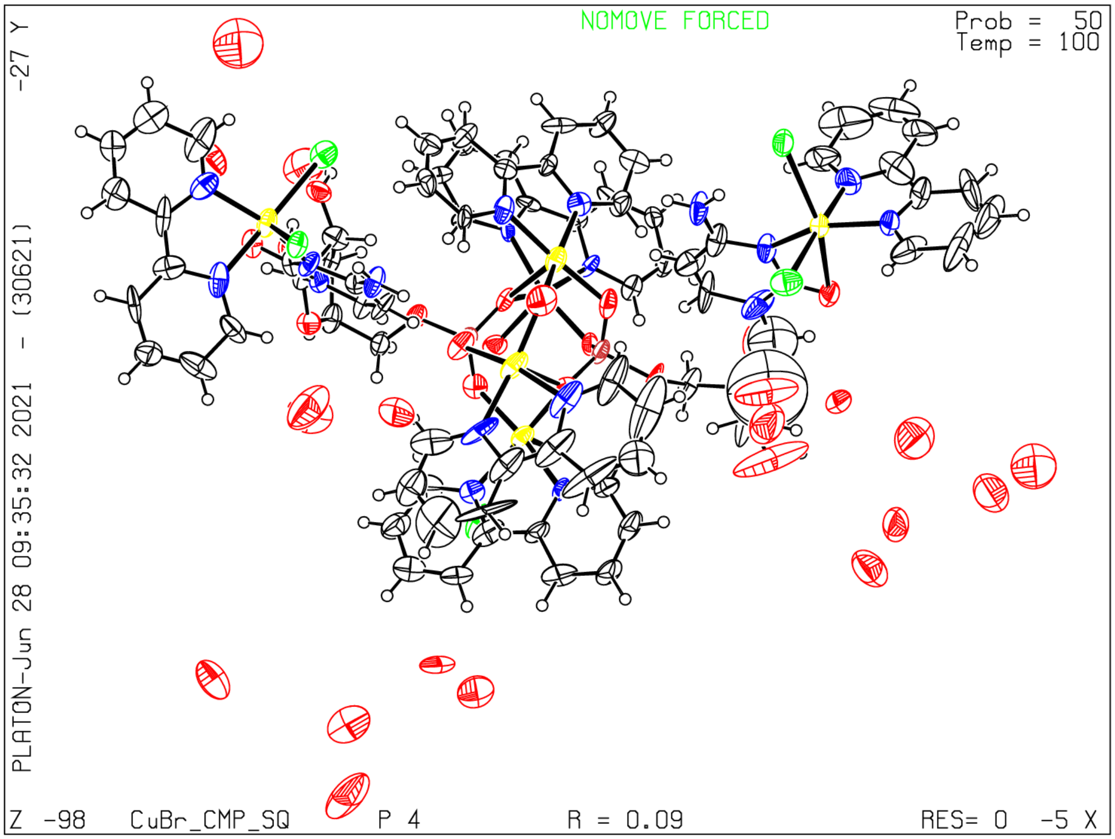

Supplement: Supplementary file 1 [file molecules-26-04594-s001.zip › CIF & CHECKCIF/CMP_MOF_1_checkcif.pdf]
